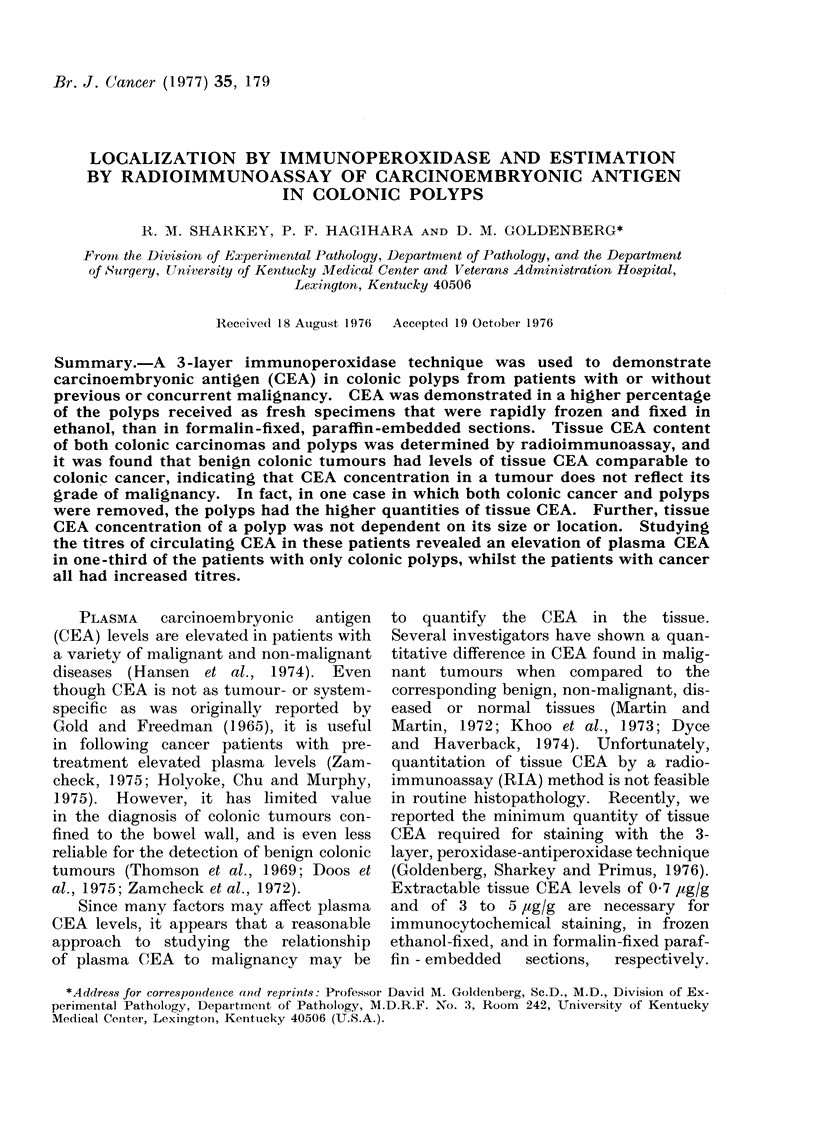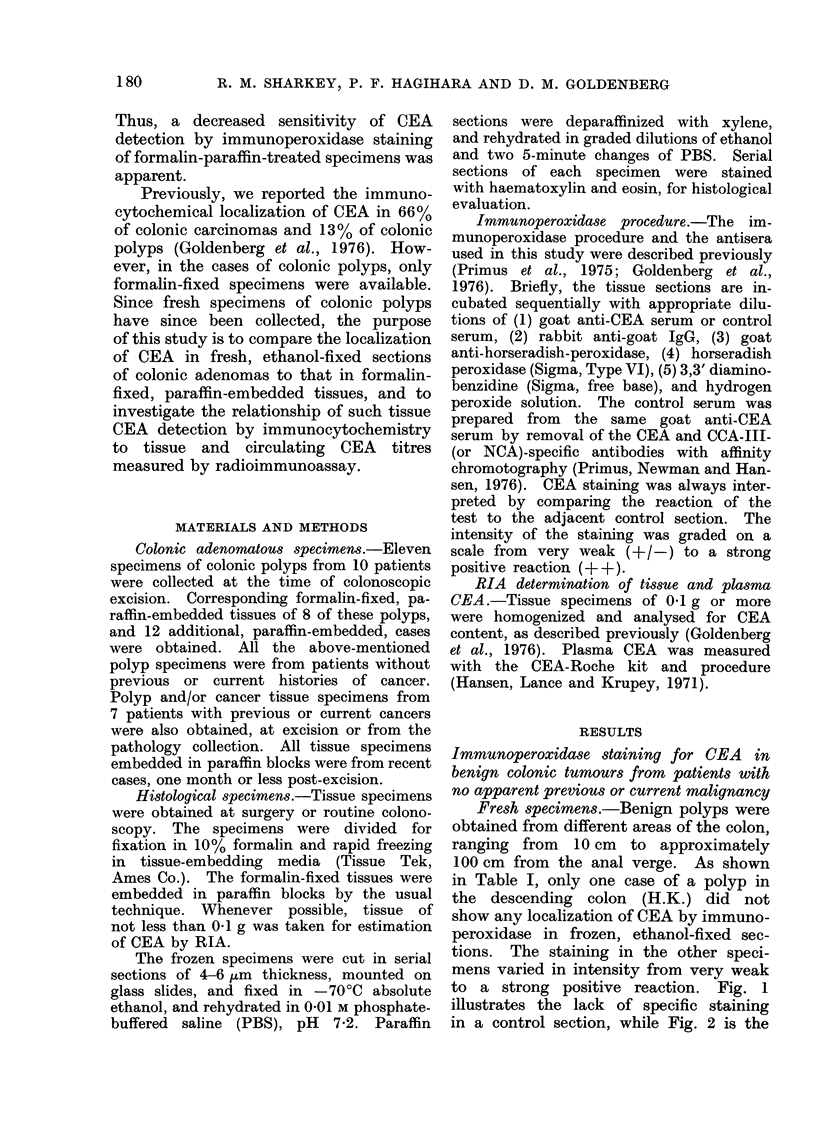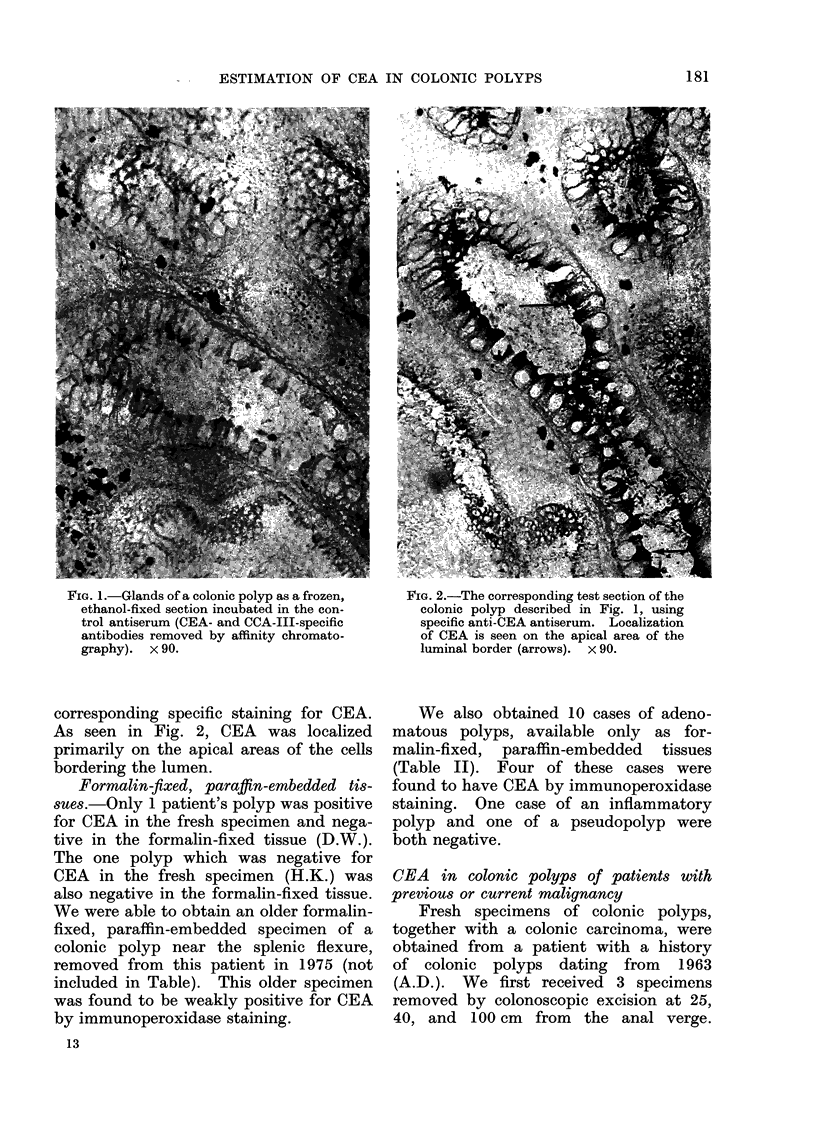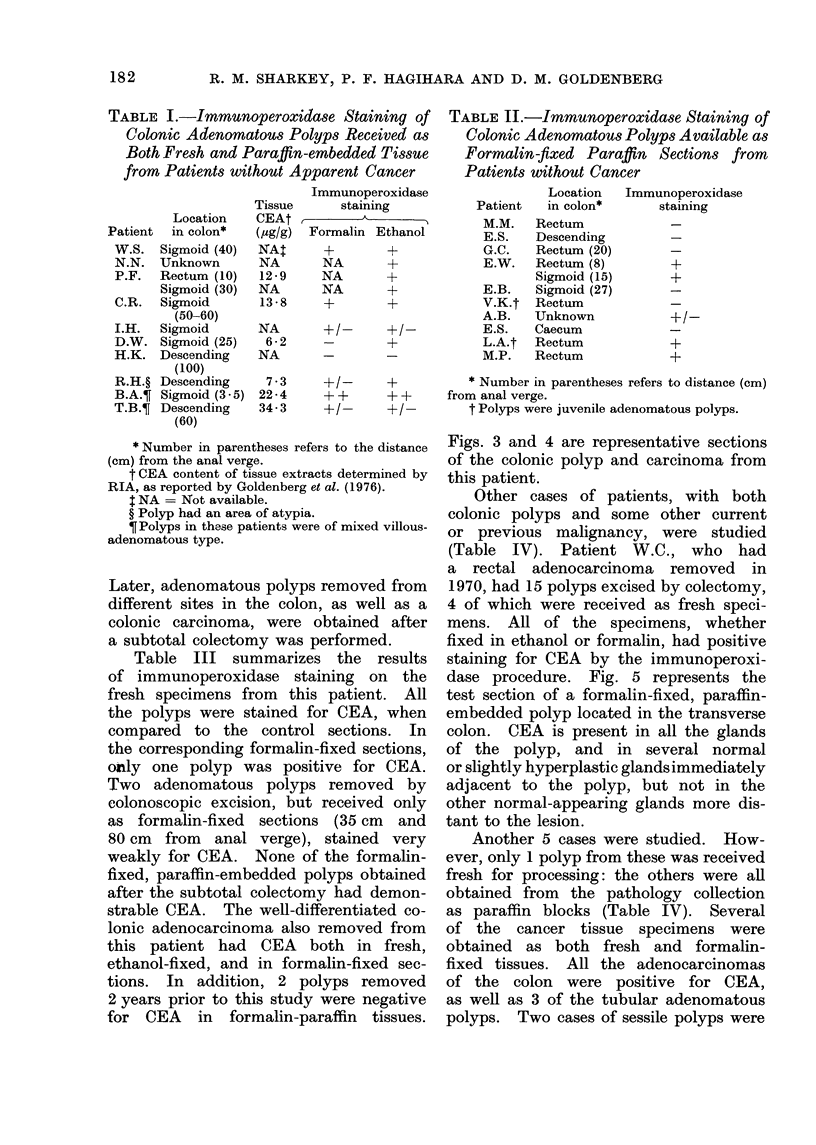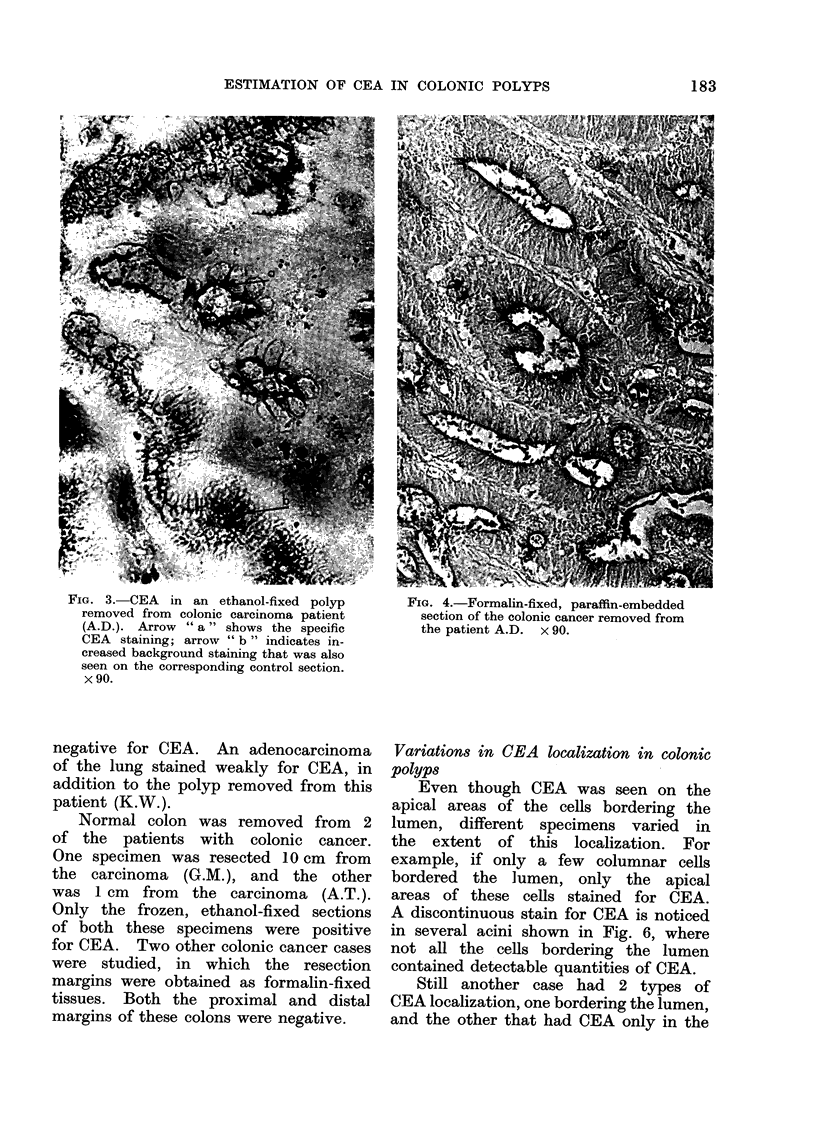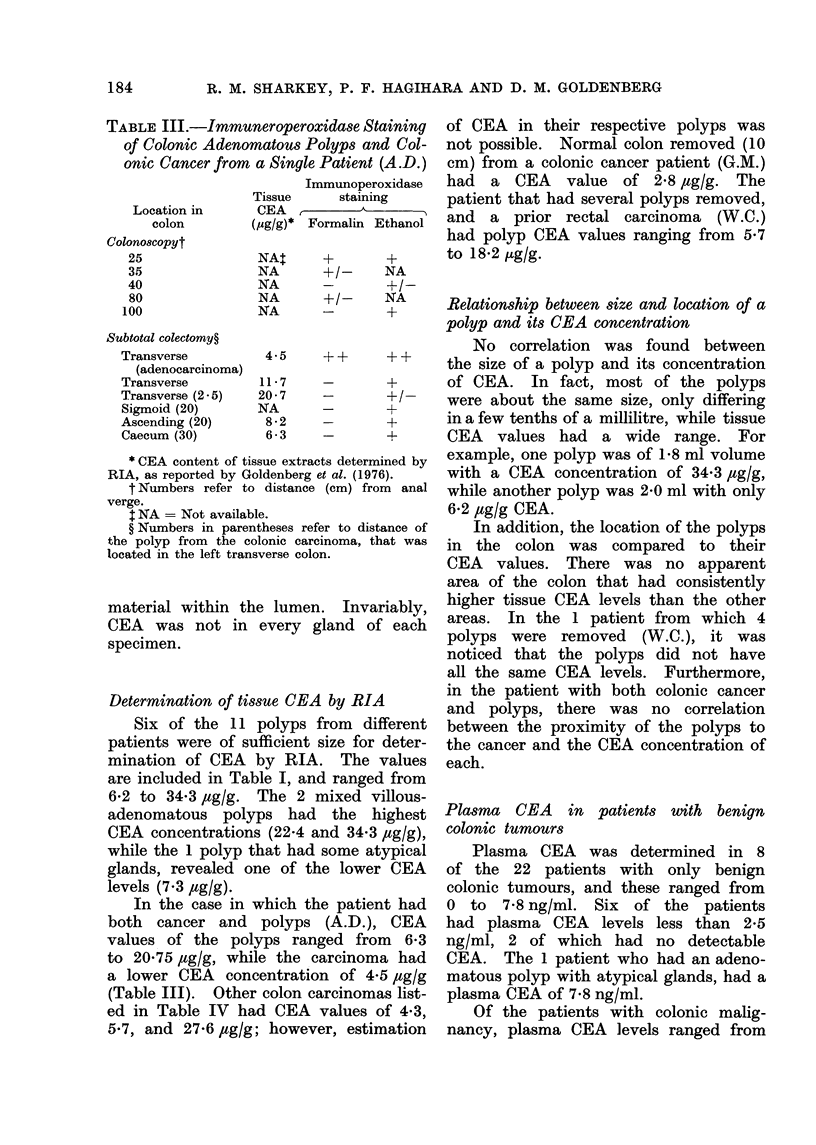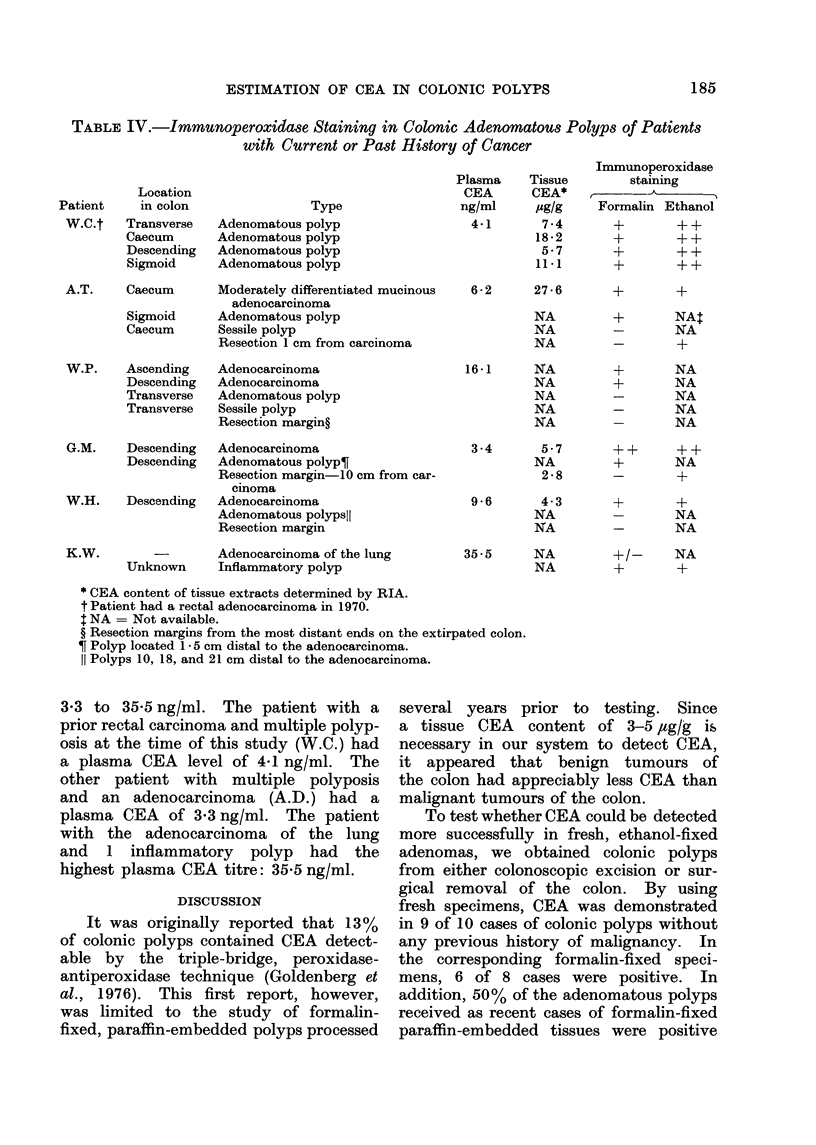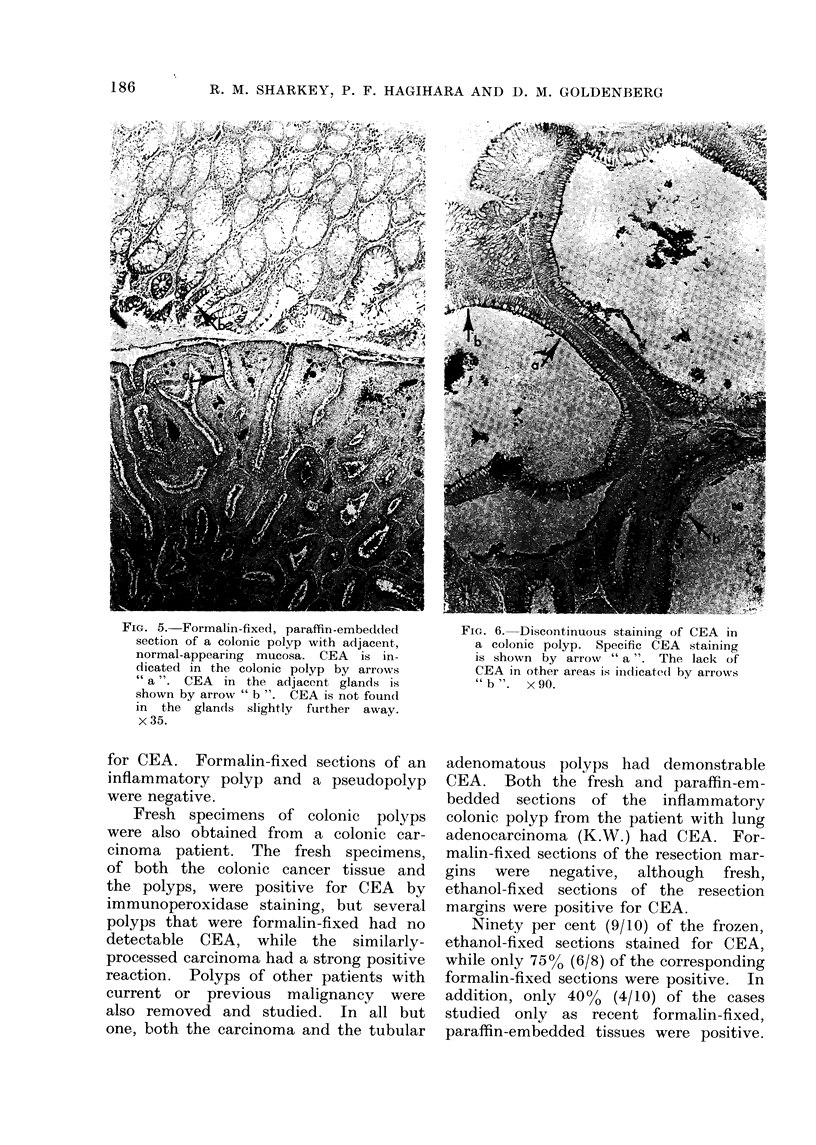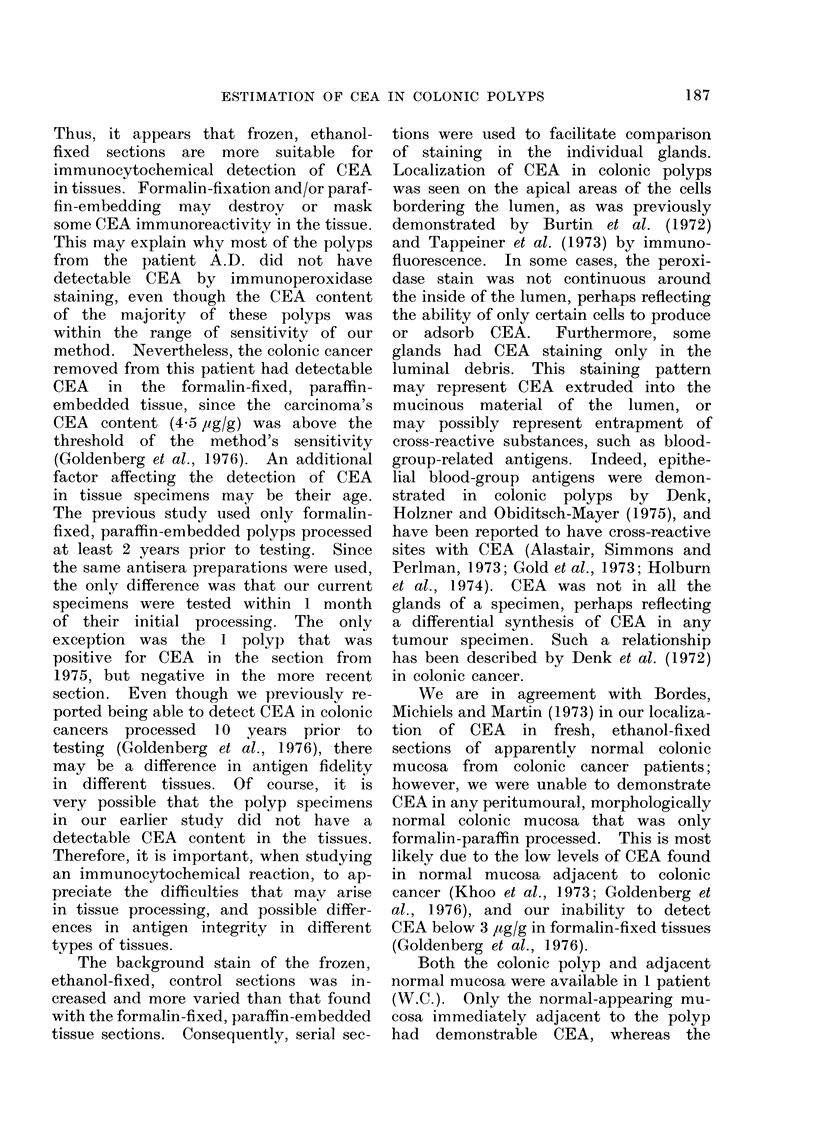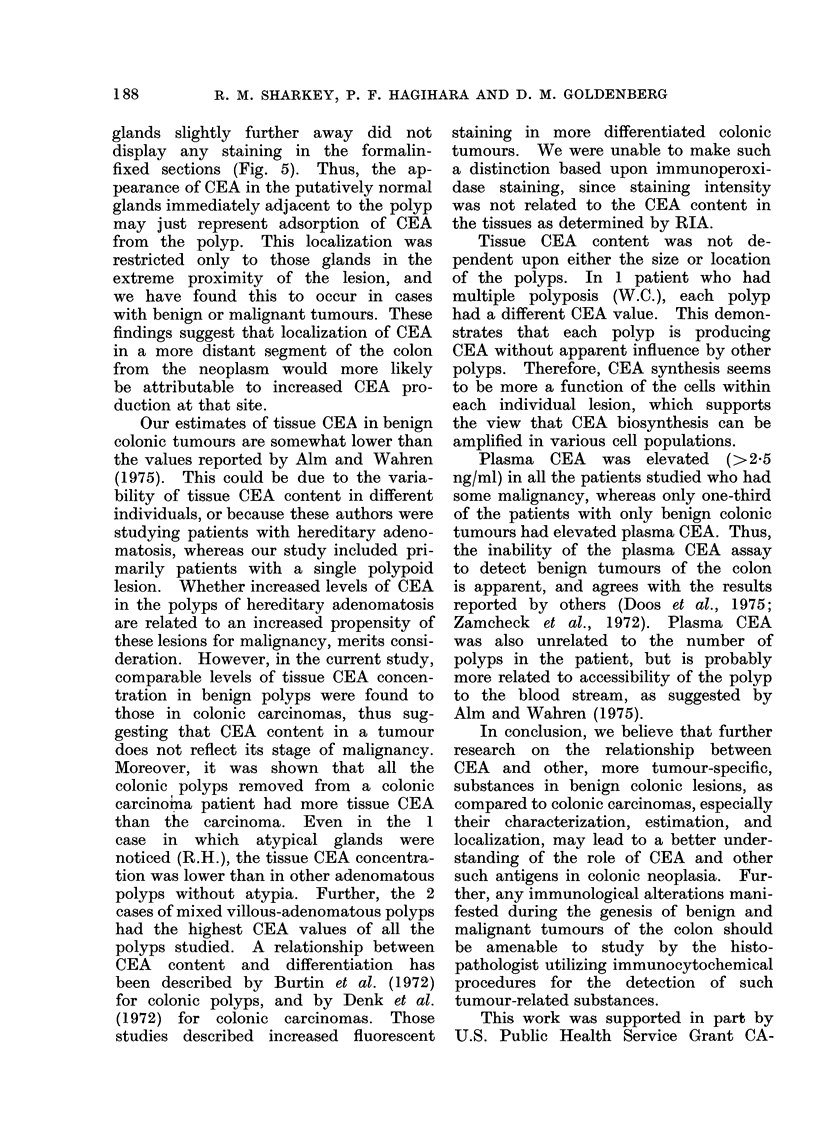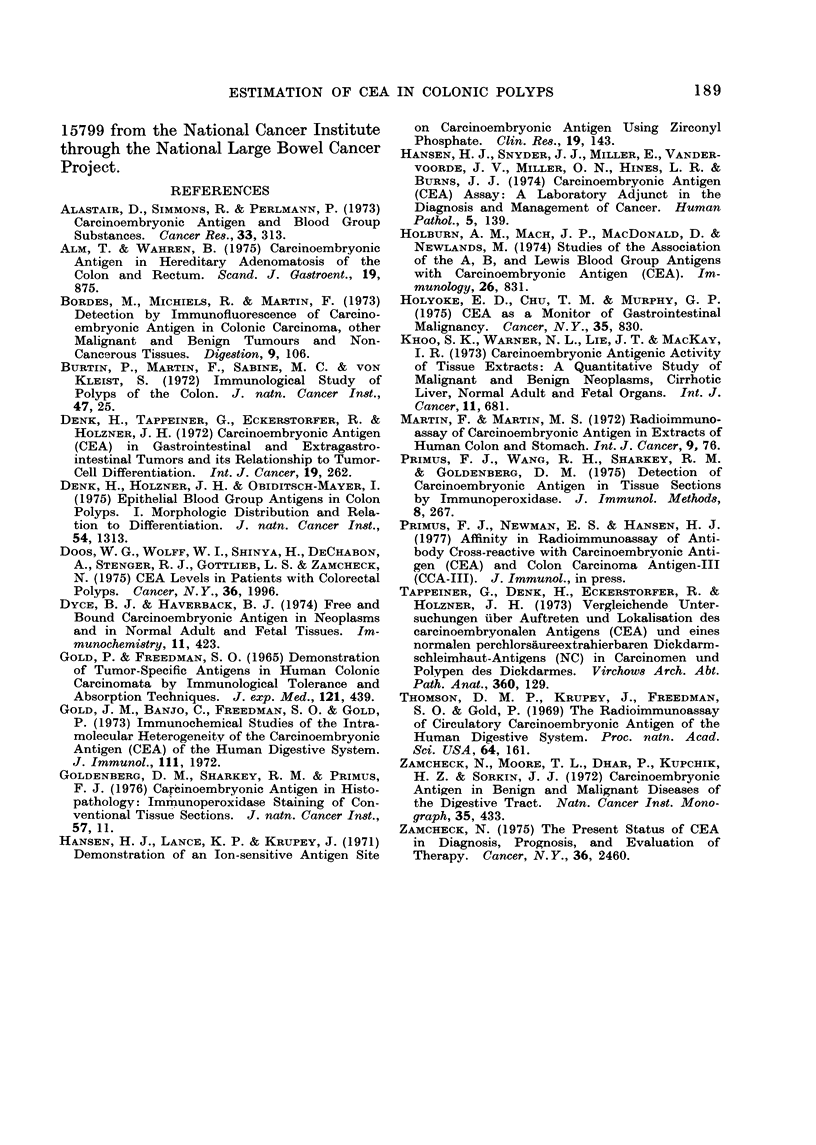# Localization by immunoperoxidase and estimation by radioimmunoassay of carcinoembryonic antigen in colonic polyps.

**DOI:** 10.1038/bjc.1977.25

**Published:** 1977-02

**Authors:** R. M. Sharkey, P. F. Hagihara, D. M. Goldenberg

## Abstract

**Images:**


*Br. J. Cancer* (1977) **35**, 179

### LOCALIZATION BY IMMUNOPEROXIDASE AND ESTIMATION BY RADIOIMMUNOASSAY OF CARCINOEMBRYONIC ANTIGEN IN COLONIC POLYPS

R. M. SHARKEY, P. F. HAGIHARA AND D. M. GOLDENBERG*

*From the Division of Experimental Pathology, Department of Pathology, and the Department of Surgery, University of Kentucky Medical Center and Veterans Administration Hospital, Lexington, Kentucky* 40506

Received 18 August 1976 Accepted 19 October 1976

**Summary.—A 3-layer immunoperoxidase technique was used to demonstrate carcinoembryonic antigen (CEA) in colonic polyps from patients with or without previous or concurrent malignancy. CEA was demonstrated in a higher percentage of the polyps received as fresh specimens that were rapidly frozen and fixed in ethanol, than in formalin-fixed, paraffin-embedded sections. Tissue CEA content of both colonic carcinomas and polyps was determined by radioimmunoassay, and it was found that benign colonic tumours had levels of tissue CEA comparable to colonic cancer, indicating that CEA concentration in a tumour does not reflect its grade of malignancy. In fact, in one case in which both colonic cancer and polyps were removed, the polyps had the higher quantities of tissue CEA. Further, tissue CEA concentration of a polyp was not dependent on its size or location. Studying the titres of circulating CEA in these patients revealed an elevation of plasma CEA in one-third of the patients with only colonic polyps, whilst the patients with cancer all had increased titres.**

PLASMA carcinoembryonic antigen (CEA) levels are elevated in patients with a variety of malignant and non-malignant diseases (Hansen *et al.*, 1974). Even though CEA is not as tumour- or system-specific as was originally reported by Gold and Freedman (1965), it is useful in following cancer patients with pre-treatment elevated plasma levels (Zamcheck, 1975; Holyoke, Chu and Murphy, 1975). However, it has limited value in the diagnosis of colonic tumours confined to the bowel wall, and is even less reliable for the detection of benign colonic tumours (Thomson *et al.*, 1969; Doos *et al.*, 1975; Zamcheck *et al.*, 1972).

Since many factors may affect plasma CEA levels, it appears that a reasonable approach to studying the relationship of plasma CEA to malignancy may be to quantify the CEA in the tissue. Several investigators have shown a quantitative difference in CEA found in malignant tumours when compared to the corresponding benign, non-malignant, diseased or normal tissues (Martin and Martin, 1972; Khoo *et al.*, 1973; Dyce and Haverback, 1974). Unfortunately, quantitation of tissue CEA by a radioimmunoassay (RIA) method is not feasible in routine histopathology. Recently, we reported the minimum quantity of tissue CEA required for staining with the 3-layer, peroxidase-antiperoxidase technique (Goldenberg, Sharkey and Primus, 1976). Extractable tissue CEA levels of 0·7 μg/g and of 3 to 5 μg/g are necessary for immunocytochemical staining, in frozen ethanol-fixed, and in formalin-fixed paraffin-embedded sections, respectively.

**Address for correspondence and reprints:* Professor David M. Goldenberg, Sc.D., M.D., Division of Experimental Pathology, Department of Pathology, M.D.R.F. No. 3, Room 242, University of Kentucky Medical Center, Lexington, Kentucky 40506 (U.S.A.).

Thus, a decreased sensitivity of CEA detection by immunoperoxidase staining of formalin-paraffin-treated specimens was apparent.

Previously, we reported the immunocytochemical localization of CEA in 66% of colonic carcinomas and 13% of colonic polyps (Goldenberg *et al.*, 1976). However, in the cases of colonic polyps, only formalin-fixed specimens were available. Since fresh specimens of colonic polyps have since been collected, the purpose of this study is to compare the localization of CEA in fresh, ethanol-fixed sections of colonic adenomas to that in formalin-fixed, paraffin-embedded tissues, and to investigate the relationship of such tissue CEA detection by immunocytochemistry to tissue and circulating CEA titres measured by radioimmunoassay.

### MATERIALS AND METHODS

*Colonic adenomatous specimens.*—Eleven specimens of colonic polyps from 10 patients were collected at the time of colonoscopic excision. Corresponding formalin-fixed, paraffin-embedded tissues of 8 of these polyps, and 12 additional, paraffin-embedded, cases were obtained. All the above-mentioned polyp specimens were from patients without previous or current histories of cancer. Polyp and/or cancer tissue specimens from 7 patients with previous or current cancers were also obtained, at excision or from the pathology collection. All tissue specimens embedded in paraffin blocks were from recent cases, one month or less post-excision.

*Histological specimens.*—Tissue specimens were obtained at surgery or routine colonoscopy. The specimens were divided for fixation in 10% formalin and rapid freezing in tissue-embedding media (Tissue Tek, Ames Co.). The formalin-fixed tissues were embedded in paraffin blocks by the usual technique. Whenever possible, tissue of not less than 0·1 g was taken for estimation of CEA by RIA.

The frozen specimens were cut in serial sections of 4–6 $\mu$m thickness, mounted on glass slides, and fixed in −70°C absolute ethanol, and rehydrated in 0·01 M phosphate-buffered saline (PBS), pH 7·2. Paraffin sections were deparaffinized with xylene, and rehydrated in graded dilutions of ethanol and two 5-minute changes of PBS. Serial sections of each specimen were stained with haematoxylin and eosin, for histological evaluation.

*Immunoperoxidase procedure.*—The immunoperoxidase procedure and the antisera used in this study were described previously (Primus *et al.*, 1975; Goldenberg *et al.*, 1976). Briefly, the tissue sections are incubated sequentially with appropriate dilutions of (1) goat anti-CEA serum or control serum, (2) rabbit anti-goat IgG, (3) goat anti-horseradish-peroxidase, (4) horseradish peroxidase (Sigma, Type VI), (5) 3,3′ diaminobenzidine (Sigma, free base), and hydrogen peroxide solution. The control serum was prepared from the same goat anti-CEA serum by removal of the CEA and CCA-III- (or NCA)-specific antibodies with affinity chromotography (Primus, Newman and Hansen, 1976). CEA staining was always interpreted by comparing the reaction of the test to the adjacent control section. The intensity of the staining was graded on a scale from very weak (+/−) to a strong positive reaction (++).

*RIA determination of tissue and plasma CEA.*—Tissue specimens of 0·1 g or more were homogenized and analysed for CEA content, as described previously (Goldenberg *et al.*, 1976). Plasma CEA was measured with the CEA-Roche kit and procedure (Hansen, Lance and Krupey, 1971).

### RESULTS

### *Immunoperoxidase staining for CEA in benign colonic tumours from patients with no apparent previous or current malignancy*

*Fresh specimens.*—Benign polyps were obtained from different areas of the colon, ranging from 10 cm to approximately 100 cm from the anal verge. As shown in Table I, only one case of a polyp in the descending colon (H.K.) did not show any localization of CEA by immunoperoxidase in frozen, ethanol-fixed sections. The staining in the other specimens varied in intensity from very weak to a strong positive reaction. Fig. 1 illustrates the lack of specific staining in a control section, while Fig. 2 is the

FIG. 1.—Glands of a colonic polyp as a frozen, ethanol-fixed section incubated in the control antiserum (CEA- and CCA-III-specific antibodies removed by affinity chromatography). ×90.

FIG. 2.—The corresponding test section of the colonic polyp described in Fig. 1, using specific anti-CEA antiserum. Localization of CEA is seen on the apical area of the luminal border (arrows). ×90.

corresponding specific staining for CEA. As seen in Fig. 2, CEA was localized primarily on the apical areas of the cells bordering the lumen.

*Formalin-fixed, paraffin-embedded tissues.*—Only 1 patient's polyp was positive for CEA in the fresh specimen and negative in the formalin-fixed tissue (D.W.). The one polyp which was negative for CEA in the fresh specimen (H.K.) was also negative in the formalin-fixed tissue. We were able to obtain an older formalin-fixed, paraffin-embedded specimen of a colonic polyp near the splenic flexure, removed from this patient in 1975 (not included in Table). This older specimen was found to be weakly positive for CEA by immunoperoxidase staining.

We also obtained 10 cases of adenomatous polyps, available only as formalin-fixed, paraffin-embedded tissues (Table II). Four of these cases were found to have CEA by immunoperoxidase staining. One case of an inflammatory polyp and one of a pseudopolyp were both negative.

*CEA in colonic polyps of patients with previous or current malignancy*

Fresh specimens of colonic polyps, together with a colonic carcinoma, were obtained from a patient with a history of colonic polyps dating from 1963 (A.D.). We first received 3 specimens removed by colonoscopic excision at 25, 40, and 100 cm from the anal verge.

TABLE I.—*Immunoperoxidase Staining of Colonic Adenomatous Polyps Received as Both Fresh and Paraffin-embedded Tissue from Patients without Apparent Cancer*

| Patient | Location in colon* | Tissue CEA† (μg/g) | Immunoperoxidase staining | |
|---|---|---|---|---|
| | | | Formalin | Ethanol |
| W.S. | Sigmoid (40) | NA‡ | + | + |
| N.N. | Unknown | NA | NA | + |
| P.F. | Rectum (10) | 12·9 | NA | + |
| | Sigmoid (30) | NA | NA | + |
| C.R. | Sigmoid (50–60) | 13·8 | + | + |
| I.H. | Sigmoid | NA | +/− | +/− |
| D.W. | Sigmoid (25) | 6·2 | − | + |
| H.K. | Descending (100) | NA | − | − |
| R.H.§ | Descending | 7·3 | +/− | + |
| B.A.¶ | Sigmoid (3·5) | 22·4 | ++ | ++ |
| T.B.¶ | Descending (60) | 34·3 | +/− | +/− |

* Number in parentheses refers to the distance (cm) from the anal verge.
† CEA content of tissue extracts determined by RIA, as reported by Goldenberg *et al.* (1976).
‡ NA = Not available.
§ Polyp had an area of atypia.
¶ Polyps in these patients were of mixed villous-adenomatous type.

Later, adenomatous polyps removed from different sites in the colon, as well as a colonic carcinoma, were obtained after a subtotal colectomy was performed.

Table III summarizes the results of immunoperoxidase staining on the fresh specimens from this patient. All the polyps were stained for CEA, when compared to the control sections. In the corresponding formalin-fixed sections, only one polyp was positive for CEA. Two adenomatous polyps removed by colonoscopic excision, but received only as formalin-fixed sections (35 cm and 80 cm from anal verge), stained very weakly for CEA. None of the formalin-fixed, paraffin-embedded polyps obtained after the subtotal colectomy had demonstrable CEA. The well-differentiated colonic adenocarcinoma also removed from this patient had CEA both in fresh, ethanol-fixed, and in formalin-fixed sections. In addition, 2 polyps removed 2 years prior to this study were negative for CEA in formalin-paraffin tissues.

TABLE II.—*Immunoperoxidase Staining of Colonic Adenomatous Polyps Available as Formalin-fixed Paraffin Sections from Patients without Cancer*

| Patient | Location in colon* | Immunoperoxidase staining |
|---|---|---|
| M.M. | Rectum | − |
| E.S. | Descending | − |
| G.C. | Rectum (20) | − |
| E.W. | Rectum (8) | + |
| | Sigmoid (15) | + |
| E.B. | Sigmoid (27) | − |
| V.K.† | Rectum | − |
| A.B. | Unknown | +/− |
| E.S. | Caecum | − |
| L.A.† | Rectum | + |
| M.P. | Rectum | + |

* Number in parentheses refers to distance (cm) from anal verge.
† Polyps were juvenile adenomatous polyps.

Figs. 3 and 4 are representative sections of the colonic polyp and carcinoma from this patient.

Other cases of patients, with both colonic polyps and some other current or previous malignancy, were studied (Table IV). Patient W.C., who had a rectal adenocarcinoma removed in 1970, had 15 polyps excised by colectomy, 4 of which were received as fresh specimens. All of the specimens, whether fixed in ethanol or formalin, had positive staining for CEA by the immunoperoxidase procedure. Fig. 5 represents the test section of a formalin-fixed, paraffin-embedded polyp located in the transverse colon. CEA is present in all the glands of the polyp, and in several normal or slightly hyperplastic glands immediately adjacent to the polyp, but not in the other normal-appearing glands more distant to the lesion.

Another 5 cases were studied. However, only 1 polyp from these was received fresh for processing: the others were all obtained from the pathology collection as paraffin blocks (Table IV). Several of the cancer tissue specimens were obtained as both fresh and formalin-fixed tissues. All the adenocarcinomas of the colon were positive for CEA, as well as 3 of the tubular adenomatous polyps. Two cases of sessile polyps were

FIG. 3.—CEA in an ethanol-fixed polyp removed from colonic carcinoma patient (A.D.). Arrow " a " shows the specific CEA staining; arrow " b " indicates increased background staining that was also seen on the corresponding control section. ×90.

FIG. 4.—Formalin-fixed, paraffin-embedded section of the colonic cancer removed from the patient A.D. ×90.

negative for CEA. An adenocarcinoma of the lung stained weakly for CEA, in addition to the polyp removed from this patient (K.W.).

Normal colon was removed from 2 of the patients with colonic cancer. One specimen was resected 10 cm from the carcinoma (G.M.), and the other was 1 cm from the carcinoma (A.T.). Only the frozen, ethanol-fixed sections of both these specimens were positive for CEA. Two other colonic cancer cases were studied, in which the resection margins were obtained as formalin-fixed tissues. Both the proximal and distal margins of these colons were negative.

*Variations in CEA localization in colonic polyps*

Even though CEA was seen on the apical areas of the cells bordering the lumen, different specimens varied in the extent of this localization. For example, if only a few columnar cells bordered the lumen, only the apical areas of these cells stained for CEA. A discontinuous stain for CEA is noticed in several acini shown in Fig. 6, where not all the cells bordering the lumen contained detectable quantities of CEA.

Still another case had 2 types of CEA localization, one bordering the lumen, and the other that had CEA only in the

TABLE III.—*Immuneroperoxidase Staining of Colonic Adenomatous Polyps and Colonic Cancer from a Single Patient (A.D.)*

| Location in colon | Tissue CEA (μg/g)* | Immunoperoxidase staining | |
|---|---|---|---|
| | | Formalin | Ethanol |
| *Colonoscopy*† | | | |
| 25 | NA‡ | + | + |
| 35 | NA | +/− | NA |
| 40 | NA | − | +/− |
| 80 | NA | +/− | NA |
| 100 | NA | − | + |
| *Subtotal colectomy*§ | | | |
| Transverse (adenocarcinoma) | 4·5 | ++ | ++ |
| Transverse | 11·7 | − | + |
| Transverse (2·5) | 20·7 | − | +/− |
| Sigmoid (20) | NA | − | + |
| Ascending (20) | 8·2 | − | + |
| Caecum (30) | 6·3 | − | + |

* CEA content of tissue extracts determined by RIA, as reported by Goldenberg *et al.* (1976).

† Numbers refer to distance (cm) from anal verge.

‡ NA = Not available.

§ Numbers in parentheses refer to distance of the polyp from the colonic carcinoma, that was located in the left transverse colon.

material within the lumen. Invariably, CEA was not in every gland of each specimen.

*Determination of tissue CEA by RIA*

Six of the 11 polyps from different patients were of sufficient size for determination of CEA by RIA. The values are included in Table I, and ranged from 6·2 to 34·3 μg/g. The 2 mixed villous-adenomatous polyps had the highest CEA concentrations (22·4 and 34·3 μg/g), while the 1 polyp that had some atypical glands, revealed one of the lower CEA levels (7·3 μg/g).

In the case in which the patient had both cancer and polyps (A.D.), CEA values of the polyps ranged from 6·3 to 20·75 μg/g, while the carcinoma had a lower CEA concentration of 4·5 μg/g (Table III). Other colon carcinomas listed in Table IV had CEA values of 4·3, 5·7, and 27·6 μg/g; however, estimation of CEA in their respective polyps was not possible. Normal colon removed (10 cm) from a colonic cancer patient (G.M.) had a CEA value of 2·8 μg/g. The patient that had several polyps removed, and a prior rectal carcinoma (W.C.) had polyp CEA values ranging from 5·7 to 18·2 μg/g.

*Relationship between size and location of a polyp and its CEA concentration*

No correlation was found between the size of a polyp and its concentration of CEA. In fact, most of the polyps were about the same size, only differing in a few tenths of a millilitre, while tissue CEA values had a wide range. For example, one polyp was of 1·8 ml volume with a CEA concentration of 34·3 μg/g, while another polyp was 2·0 ml with only 6·2 μg/g CEA.

In addition, the location of the polyps in the colon was compared to their CEA values. There was no apparent area of the colon that had consistently higher tissue CEA levels than the other areas. In the 1 patient from which 4 polyps were removed (W.C.), it was noticed that the polyps did not have all the same CEA levels. Furthermore, in the patient with both colonic cancer and polyps, there was no correlation between the proximity of the polyps to the cancer and the CEA concentration of each.

*Plasma CEA in patients with benign colonic tumours*

Plasma CEA was determined in 8 of the 22 patients with only benign colonic tumours, and these ranged from 0 to 7·8 ng/ml. Six of the patients had plasma CEA levels less than 2·5 ng/ml, 2 of which had no detectable CEA. The 1 patient who had an adenomatous polyp with atypical glands, had a plasma CEA of 7·8 ng/ml.

Of the patients with colonic malignancy, plasma CEA levels ranged from

TABLE IV.—*Immunoperoxidase Staining in Colonic Adenomatous Polyps of Patients with Current or Past History of Cancer*

| Patient | Location in colon | Type | Plasma CEA ng/ml | Tissue CEA* μg/g | Immunoperoxidase staining Formalin | Ethanol |
|---|---|---|---|---|---|---|
| W.C.† | Transverse | Adenomatous polyp | 4·1 | 7·4 | + | ++ |
| | Caecum | Adenomatous polyp | | 18·2 | + | ++ |
| | Descending | Adenomatous polyp | | 5·7 | + | ++ |
| | Sigmoid | Adenomatous polyp | | 11·1 | + | ++ |
| A.T. | Caecum | Moderately differentiated mucinous adenocarcinoma | 6·2 | 27·6 | + | + |
| | Sigmoid | Adenomatous polyp | | NA | + | NA‡ |
| | Caecum | Sessile polyp | | NA | − | NA |
| | | Resection 1 cm from carcinoma | | NA | − | + |
| W.P. | Ascending | Adenocarcinoma | 16·1 | NA | + | NA |
| | Descending | Adenocarcinoma | | NA | + | NA |
| | Transverse | Adenomatous polyp | | NA | − | NA |
| | Transverse | Sessile polyp | | NA | − | NA |
| | | Resection margin§ | | NA | − | NA |
| G.M. | Descending | Adenocarcinoma | 3·4 | 5·7 | ++ | ++ |
| | Descending | Adenomatous polyp¶ | | NA | + | NA |
| | | Resection margin—10 cm from carcinoma | | 2·8 | − | + |
| W.H. | Descending | Adenocarcinoma | 9·6 | 4·3 | + | + |
| | | Adenomatous polyps‖ | | NA | − | NA |
| | | Resection margin | | NA | − | NA |
| K.W. | — | Adenocarcinoma of the lung | 35·5 | NA | +/− | NA |
| | Unknown | Inflammatory polyp | | NA | + | + |

* CEA content of tissue extracts determined by RIA.
† Patient had a rectal adenocarcinoma in 1970.
‡ NA = Not available.
§ Resection margins from the most distant ends on the extirpated colon.
¶ Polyp located 1·5 cm distal to the adenocarcinoma.
‖ Polyps 10, 18, and 21 cm distal to the adenocarcinoma.

3·3 to 35·5 ng/ml. The patient with a prior rectal carcinoma and multiple polyposis at the time of this study (W.C.) had a plasma CEA level of 4·1 ng/ml. The other patient with multiple polyposis and an adenocarcinoma (A.D.) had a plasma CEA of 3·3 ng/ml. The patient with the adenocarcinoma of the lung and 1 inflammatory polyp had the highest plasma CEA titre: 35·5 ng/ml.

### DISCUSSION

It was originally reported that 13% of colonic polyps contained CEA detectable by the triple-bridge, peroxidase-antiperoxidase technique (Goldenberg *et al.*, 1976). This first report, however, was limited to the study of formalin-fixed, paraffin-embedded polyps processed several years prior to testing. Since a tissue CEA content of 3–5 μg/g is necessary in our system to detect CEA, it appeared that benign tumours of the colon had appreciably less CEA than malignant tumours of the colon.

To test whether CEA could be detected more successfully in fresh, ethanol-fixed adenomas, we obtained colonic polyps from either colonoscopic excision or surgical removal of the colon. By using fresh specimens, CEA was demonstrated in 9 of 10 cases of colonic polyps without any previous history of malignancy. In the corresponding formalin-fixed specimens, 6 of 8 cases were positive. In addition, 50% of the adenomatous polyps received as recent cases of formalin-fixed paraffin-embedded tissues were positive

FIG. 5.—Formalin-fixed, paraffin-embedded section of a colonic polyp with adjacent, normal-appearing mucosa. CEA is indicated in the colonic polyp by arrows "a". CEA in the adjacent glands is shown by arrow "b". CEA is not found in the glands slightly further away. ×35.

FIG. 6.—Discontinuous staining of CEA in a colonic polyp. Specific CEA staining is shown by arrow "a". The lack of CEA in other areas is indicated by arrows "b". ×90.

for CEA. Formalin-fixed sections of an inflammatory polyp and a pseudopolyp were negative.

Fresh specimens of colonic polyps were also obtained from a colonic carcinoma patient. The fresh specimens, of both the colonic cancer tissue and the polyps, were positive for CEA by immunoperoxidase staining, but several polyps that were formalin-fixed had no detectable CEA, while the similarly-processed carcinoma had a strong positive reaction. Polyps of other patients with current or previous malignancy were also removed and studied. In all but one, both the carcinoma and the tubular adenomatous polyps had demonstrable CEA. Both the fresh and paraffin-embedded sections of the inflammatory colonic polyp from the patient with lung adenocarcinoma (K.W.) had CEA. Formalin-fixed sections of the resection margins were negative, although fresh, ethanol-fixed sections of the resection margins were positive for CEA.

Ninety per cent (9/10) of the frozen, ethanol-fixed sections stained for CEA, while only 75% (6/8) of the corresponding formalin-fixed sections were positive. In addition, only 40% (4/10) of the cases studied only as recent formalin-fixed, paraffin-embedded tissues were positive.

Thus, it appears that frozen, ethanol-fixed sections are more suitable for immunocytochemical detection of CEA in tissues. Formalin-fixation and/or paraffin-embedding may destroy or mask some CEA immunoreactivity in the tissue. This may explain why most of the polyps from the patient A.D. did not have detectable CEA by immunoperoxidase staining, even though the CEA content of the majority of these polyps was within the range of sensitivity of our method. Nevertheless, the colonic cancer removed from this patient had detectable CEA in the formalin-fixed, paraffin-embedded tissue, since the carcinoma's CEA content (4·5 $\mu$g/g) was above the threshold of the method's sensitivity (Goldenberg *et al.*, 1976). An additional factor affecting the detection of CEA in tissue specimens may be their age. The previous study used only formalin-fixed, paraffin-embedded polyps processed at least 2 years prior to testing. Since the same antisera preparations were used, the only difference was that our current specimens were tested within 1 month of their initial processing. The only exception was the 1 polyp that was positive for CEA in the section from 1975, but negative in the more recent section. Even though we previously reported being able to detect CEA in colonic cancers processed 10 years prior to testing (Goldenberg *et al.*, 1976), there may be a difference in antigen fidelity in different tissues. Of course, it is very possible that the polyp specimens in our earlier study did not have a detectable CEA content in the tissues. Therefore, it is important, when studying an immunocytochemical reaction, to appreciate the difficulties that may arise in tissue processing, and possible differences in antigen integrity in different types of tissues.

The background stain of the frozen, ethanol-fixed, control sections was increased and more varied than that found with the formalin-fixed, paraffin-embedded tissue sections. Consequently, serial sections were used to facilitate comparison of staining in the individual glands. Localization of CEA in colonic polyps was seen on the apical areas of the cells bordering the lumen, as was previously demonstrated by Burtin *et al.* (1972) and Tappeiner *et al.* (1973) by immunofluorescence. In some cases, the peroxidase stain was not continuous around the inside of the lumen, perhaps reflecting the ability of only certain cells to produce or adsorb CEA. Furthermore, some glands had CEA staining only in the luminal debris. This staining pattern may represent CEA extruded into the mucinous material of the lumen, or may possibly represent entrapment of cross-reactive substances, such as blood-group-related antigens. Indeed, epithelial blood-group antigens were demonstrated in colonic polyps by Denk, Holzner and Obiditsch-Mayer (1975), and have been reported to have cross-reactive sites with CEA (Alastair, Simmons and Perlman, 1973; Gold *et al.*, 1973; Holburn *et al.*, 1974). CEA was not in all the glands of a specimen, perhaps reflecting a differential synthesis of CEA in any tumour specimen. Such a relationship has been described by Denk *et al.* (1972) in colonic cancer.

We are in agreement with Bordes, Michiels and Martin (1973) in our localization of CEA in fresh, ethanol-fixed sections of apparently normal colonic mucosa from colonic cancer patients; however, we were unable to demonstrate CEA in any peritumoural, morphologically normal colonic mucosa that was only formalin-paraffin processed. This is most likely due to the low levels of CEA found in normal mucosa adjacent to colonic cancer (Khoo *et al.*, 1973; Goldenberg *et al.*, 1976), and our inability to detect CEA below 3 $\mu$g/g in formalin-fixed tissues (Goldenberg *et al.*, 1976).

Both the colonic polyp and adjacent normal mucosa were available in 1 patient (W.C.). Only the normal-appearing mucosa immediately adjacent to the polyp had demonstrable CEA, whereas the glands slightly further away did not display any staining in the formalin-fixed sections (Fig. 5). Thus, the appearance of CEA in the putatively normal glands immediately adjacent to the polyp may just represent adsorption of CEA from the polyp. This localization was restricted only to those glands in the extreme proximity of the lesion, and we have found this to occur in cases with benign or malignant tumours. These findings suggest that localization of CEA in a more distant segment of the colon from the neoplasm would more likely be attributable to increased CEA production at that site.

Our estimates of tissue CEA in benign colonic tumours are somewhat lower than the values reported by Alm and Wahren (1975). This could be due to the variability of tissue CEA content in different individuals, or because these authors were studying patients with hereditary adenomatosis, whereas our study included primarily patients with a single polypoid lesion. Whether increased levels of CEA in the polyps of hereditary adenomatosis are related to an increased propensity of these lesions for malignancy, merits consideration. However, in the current study, comparable levels of tissue CEA concentration in benign polyps were found to those in colonic carcinomas, thus suggesting that CEA content in a tumour does not reflect its stage of malignancy. Moreover, it was shown that all the colonic polyps removed from a colonic carcinoma patient had more tissue CEA than the carcinoma. Even in the 1 case in which atypical glands were noticed (R.H.), the tissue CEA concentration was lower than in other adenomatous polyps without atypia. Further, the 2 cases of mixed villous-adenomatous polyps had the highest CEA values of all the polyps studied. A relationship between CEA content and differentiation has been described by Burtin *et al.* (1972) for colonic polyps, and by Denk *et al.* (1972) for colonic carcinomas. Those studies described increased fluorescent staining in more differentiated colonic tumours. We were unable to make such a distinction based upon immunoperoxidase staining, since staining intensity was not related to the CEA content in the tissues as determined by RIA.

Tissue CEA content was not dependent upon either the size or location of the polyps. In 1 patient who had multiple polyposis (W.C.), each polyp had a different CEA value. This demonstrates that each polyp is producing CEA without apparent influence by other polyps. Therefore, CEA synthesis seems to be more a function of the cells within each individual lesion, which supports the view that CEA biosynthesis can be amplified in various cell populations.

Plasma CEA was elevated (>2·5 ng/ml) in all the patients studied who had some malignancy, whereas only one-third of the patients with only benign colonic tumours had elevated plasma CEA. Thus, the inability of the plasma CEA assay to detect benign tumours of the colon is apparent, and agrees with the results reported by others (Doos *et al.*, 1975; Zamcheck *et al.*, 1972). Plasma CEA was also unrelated to the number of polyps in the patient, but is probably more related to accessibility of the polyp to the blood stream, as suggested by Alm and Wahren (1975).

In conclusion, we believe that further research on the relationship between CEA and other, more tumour-specific, substances in benign colonic lesions, as compared to colonic carcinomas, especially their characterization, estimation, and localization, may lead to a better understanding of the role of CEA and other such antigens in colonic neoplasia. Further, any immunological alterations manifested during the genesis of benign and malignant tumours of the colon should be amenable to study by the histopathologist utilizing immunocytochemical procedures for the detection of such tumour-related substances.

This work was supported in part by U.S. Public Health Service Grant CA-

15799 from the National Cancer Institute through the National Large Bowel Cancer Project.

REFERENCES

ALASTAIR, D., SIMMONS, R. & PERLMANN, P. (1973) Carcinoembryonic Antigen and Blood Group Substances. *Cancer Res.*, **33**, 313.

ALM, T. & WAHREN, B. (1975) Carcinoembryonic Antigen in Hereditary Adenomatosis of the Colon and Rectum. *Scand. J. Gastroent.*, **19**, 875.

BORDES, M., MICHIELS, R. & MARTIN, F. (1973) Detection by Immunofluorescence of Carcinoembryonic Antigen in Colonic Carcinoma, other Malignant and Benign Tumours and Non-Cancerous Tissues. *Digestion*, **9**, 106.

BURTIN, P., MARTIN, F., SABINE, M. C. & VON KLEIST, S. (1972) Immunological Study of Polyps of the Colon. *J. natn. Cancer Inst.*, **47**, 25.

DENK, H., TAPPEINER, G., ECKERSTORFER, R. & HOLZNER, J. H. (1972) Carcinoembryonic Antigen (CEA) in Gastrointestinal and Extragastrointestinal Tumors and its Relationship to Tumor-Cell Differentiation. *Int. J. Cancer*, **19**, 262.

DENK, H., HOLZNER, J. H. & OBIDITSCH-MAYER, I. (1975) Epithelial Blood Group Antigens in Colon Polyps. I. Morphologic Distribution and Relation to Differentiation. *J. natn. Cancer Inst.*, **54**, 1313.

DOOS, W. G., WOLFF, W. I., SHINYA, H., DECHABON, A., STENGER, R. J., GOTTLIEB, L. S. & ZAMCHECK, N. (1975) CEA Levels in Patients with Colorectal Polyps. *Cancer, N.Y.*, **36**, 1996.

DYCE, B. J. & HAVERBACK, B. J. (1974) Free and Bound Carcinoembryonic Antigen in Neoplasms and in Normal Adult and Fetal Tissues. *Immunochemistry*, **11**, 423.

GOLD, P. & FREEDMAN, S. O. (1965) Demonstration of Tumor-Specific Antigens in Human Colonic Carcinomata by Immunological Tolerance and Absorption Techniques. *J. exp. Med.*, **121**, 439.

GOLD, J. M., BANJO, C., FREEDMAN, S. O. & GOLD, P. (1973) Immunochemical Studies of the Intramolecular Heterogeneity of the Carcinoembryonic Antigen (CEA) of the Human Digestive System. *J. Immunol.*, **111**, 1972.

GOLDENBERG, D. M., SHARKEY, R. M. & PRIMUS, F. J. (1976) Carcinoembryonic Antigen in Histopathology: Immunoperoxidase Staining of Conventional Tissue Sections. *J. natn. Cancer Inst.*, **57**, 11.

HANSEN, H. J., LANCE, K. P. & KRUPEY, J. (1971) Demonstration of an Ion-sensitive Antigen Site on Carcinoembryonic Antigen Using Zirconyl Phosphate. *Clin. Res.*, **19**, 143.

HANSEN, H. J., SNYDER, J. J., MILLER, E., VANDERVOORDE, J. V., MILLER, O. N., HINES, L. R. & BURNS, J. J. (1974) Carcinoembryonic Antigen (CEA) Assay: A Laboratory Adjunct in the Diagnosis and Management of Cancer. *Human Pathol.*, **5**, 139.

HOLBURN, A. M., MACH, J. P., MACDONALD, D. & NEWLANDS, M. (1974) Studies of the Association of the A, B, and Lewis Blood Group Antigens with Carcinoembryonic Antigen (CEA). *Immunology*, **26**, 831.

HOLYOKE, E. D., CHU, T. M. & MURPHY, G. P. (1975) CEA as a Monitor of Gastrointestinal Malignancy. *Cancer, N.Y.*, **35**, 830.

KHOO, S. K., WARNER, N. L., LIE, J. T. & MACKAY, I. R. (1973) Carcinoembryonic Antigenic Activity of Tissue Extracts: A Quantitative Study of Malignant and Benign Neoplasms, Cirrhotic Liver, Normal Adult and Fetal Organs. *Int. J. Cancer*, **11**, 681.

MARTIN, F. & MARTIN, M. S. (1972) Radioimmunoassay of Carcinoembryonic Antigen in Extracts of Human Colon and Stomach. *Int. J. Cancer*, **9**, 76.

PRIMUS, F. J., WANG, R. H., SHARKEY, R. M. & GOLDENBERG, D. M. (1975) Detection of Carcinoembryonic Antigen in Tissue Sections by Immunoperoxidase. *J. Immunol. Methods*, **8**, 267.

PRIMUS, F. J., NEWMAN, E. S. & HANSEN, H. J. (1977) Affinity in Radioimmunoassay of Antibody Cross-reactive with Carcinoembryonic Antigen (CEA) and Colon Carcinoma Antigen-III (CCA-III). *J. Immunol.*, in press.

TAPPEINER, G., DENK, H., ECKERSTORFER, R. & HOLZNER, J. H. (1973) Vergleichende Untersuchungen über Auftreten und Lokalisation des carcinoembryonalen Antigens (CEA) und eines normalen perchlorsäureextrahierbaren Dickdarmschleimhaut-Antigens (NC) in Carcinomen und Polypen des Dickdarmes. *Virchows Arch. Abt. Path. Anat.*, **360**, 129.

THOMSON, D. M. P., KRUPEY, J., FREEDMAN, S. O. & Gold, P. (1969) The Radioimmunoassay of Circulatory Carcinoembryonic Antigen of the Human Digestive System. *Proc. natn. Acad. Sci. USA*, **64**, 161.

ZAMCHECK, N., MOORE, T. L., DHAR, P., KUPCHIK, H. Z. & SORKIN, J. J. (1972) Carcinoembryonic Antigen in Benign and Malignant Diseases of the Digestive Tract. *Natn. Cancer Inst. Monograph*, **35**, 433.

ZAMCHECK, N. (1975) The Present Status of CEA in Diagnosis, Prognosis, and Evaluation of Therapy. *Cancer, N.Y.*, **36**, 2460.